# Strategies for coping and dealing with lateral violence among Aboriginal people living in south-east Australia

**DOI:** 10.1080/00049530.2024.2347646

**Published:** 2024-05-12

**Authors:** Theoni Whyman, Cammi Murrup-Stewart, Uncle Michael Young, Adrian Carter, Laura Jobson

**Affiliations:** a Turner Institute for Brain and Mental Health and School of Psychological Sciences, Monash University, Clayton, Victoria, Australia; b Telethon Kids Institute, University of Western Australia, Perth, Western Australia, Australia; c Aboriginal Community Elder, Mildura, Australia

**Keywords:** Lateral violence, Aboriginal health, coping strategies, social and emotional wellbeing, identity, qualitative research

## Abstract

**Objective:**

Lateral violence, a group of behaviours directed towards people of the same group, is considered endemic among Aboriginal people. Behaviours include bullying, gossiping, isolation or exclusion of certain group members, and challenges to one’s Aboriginal identity. Lateral violence impacts all aspects of one’s life. Due to its pervasiveness, this qualitative study investigated strategies employed by Aboriginal people to deal with lateral violence.

**Method:**

Yarns with 17 knowledge-holders (53% male, 47% female; aged between 18 and 65 years) took place in south-east Australia. Thematic analysis and yarn summaries were used to analyse the yarns.

**Results:**

Strategies identified to deal with lateral violence included changes to one’s attitude towards lateral violence, connecting with others, one’s culture and community to improve wellbeing, education strategies, and systemic change.

**Conclusion:**

Supporting the development of Aboriginal identity, promoting social and emotional wellbeing, and true self-determination has the potential to heal Aboriginal communities and reduce lateral violence in the process.

## Key Points

**What is already known about this topic:**

1. Lateral violence refers to a group of overt and covert behaviours including bullying, gossiping, family feuding, workplace conflict, and isolating or excluding certain members of the community.
2. Lateral violence has recently been extended to encompass attacks on one’s Indigeneity and identity.
3. Lateral violence not only impacts the targeted individual, but also one’s family, friends, and community.

**What this topic adds:**

1. Aboriginal people employ many strategies to cope, and deal, with lateral violence.
2. A strong sense of one’s Indigeneity was important for overcoming challenges to one’s identity.
3. Systemic change, including a focus on Aboriginal self-determination, is important for SEWB and overcoming lateral violence.

## Introduction

Still today, Indigenous people around the world continue to endure the impacts of colonisation and oppression. This is due to the fact that colonisation is not a discrete historical event. Rather it is a system, or structure, set up in the colonised land to benefit the colonisers (Wolfe, 2006). The bias inherent in this colonial system continues to oppress Indigenous peoples (Wolfe, 2006). Following invasion, Australia became a settler-colonial state which persists to this day (Tuck & Yang, 2012). Therefore, issues stemming from colonisation and the settler-colonial state cannot be resolved while these systems continue. Lateral violence, a group of behaviours enacted on others of the same group, is a problem endemic to many Indigenous peoples (Frankland, 2008). It is thought to be a result of living in a settler-colonial system (where settlers come to a new land and settle, such as occurred in Australia) (Australian Human Rights Commission [AHRC] 2011; Whyman et al., 2021). This paper investigates the ways Aboriginal people, a group of Indigenous people in Australia, cope and deal with the lateral violence they experience.

Lateral violence refers to a group of overt and covert behaviours including bullying, gossiping, family feuding, workplace conflict, and isolating or excluding certain members of the community (AHRC, 2011). Lateral violence also extends to include attacks on one’s Indigeneity and identity (Clark et al., 2016; Whyman et al., 2022). Lateral violence “occurs when, out of anger and frustration, an oppressed group turns on itself and begins to violate each other” rather than the oppressor (Mohawk Rod Jefferies in Frankland, 2008, p. 23). Lateral violence stems from the oppression of one group over another (Fanon, 1963; Freire, 1970; Ture & Hamilton, 1967). The oppressing group holds certain beliefs and values about their own group and the group they oppress. Following generations of oppression, these often negative beliefs and values can become internalised by the oppressed group (Memmi, 1965). This is known as internalised oppression (David & Derthick, 2014; Freire, 1970) and consists of feelings such as hatred of oneself, inferiority, isolation, powerlessness, and feelings of “gratefulness [to the oppressor] for being allowed to survive” (Pheterson, 1986, p. 148). These negative emotions, when expressed towards oneself or others in the oppressed group, is known as lateral violence (Cripps & Adams, 2014; Fanon, 1963; Freire, 1970). Attacks on another’s Indigenous identity are common. This arises due to issues or struggles with one’s own identity, stemming from settler colonisers introduced notions of an authentic Indigenous person and Indigenous identity (Gorringe et al., 2011). In Australia, terms such as “full-blood”, “half-caste”, “octoroon” (based on the presence or lack of certain pre-colonial Aboriginal physical features) were used by settler colonisers to undermine Aboriginal status (Human Rights and Equal Opportunity Commission, 1997). This was used to make it seem as though Aboriginal people had disappeared in order to gain access to their land (Tuck & Yang, 2012). The internalisation of settler colonial notions of authentic Aboriginal identity has led many Aboriginal people to police Aboriginal identity, based on those notions or stereotypes (Gorringe et al., 2011). Here, the oppressed continue the work of the oppressor by oppressing each other (Fanon, 1963).

The impacts of lateral violence are felt in all aspects of one’s life. Impacts include poor self-esteem, stress, sleep disorders, depression, and anxiety (Bennett, 2014; Clark & Augoustinos, 2015). Workplace lateral violence can lead to decreased productivity, increased absenteeism, and high staff turnover (Namie, 2008). In Australia, research has found that lateral violence negatively impacts Aboriginal people’s sense of Aboriginality and their wellbeing (Clark et al., 2016; Doyle et al., 2017; Whyman et al., 2022). Clark et al. (2016) described lateral violence as a “soul destroying bug” that strips away at one’s Aboriginality (p. 49). These intra-racial attacks on identity are thought to be more severe than inter-racial attacks (Coffin, 2011; Stoor et al., 2019) that can lead individuals to question their Indigeneity, feel ashamed of their heritage (Bennett, 2014; Clark et al., 2016), and not feel Indigenous enough (Doyle et al., 2017). As a result, lateral violence can cause some individuals to disengage from their Aboriginal community and culture, increasing the loss of cultural knowledge (Clark et al., 2016; Whyman et al., 2022). Lateral violence not only impacts the targeted individual, but also one’s family, friends, and community (Sheridan-Leos, 2008; Whyman et al., 2022).

Considering the significant impacts lateral violence has on the wellbeing of Indigenous peoples, it is important to understand the strategies Indigenous people employ to deal with lateral violence. To date, a study by Clark et al. (2017) is the only study that has explicitly examined coping strategies in response to lateral violence among Aboriginal people. That study explored the coping strategies of 30 Aboriginal people, primarily women between 41 to 50 years of age, in Adelaide, Australia. Participants believed education and raising awareness of lateral violence to be a central strategy. Some also noted that they sought support when experiencing lateral violence, most commonly as social and family support, support in the workplace, legal support, and professional counselling. Some avoided interacting with other Aboriginal people who perpetrate lateral violence, including their family members, communities, and Aboriginal organisations. Others drew inspiration and strength from their culture in building their sense of Aboriginality, while others discussed the importance of role models and champions in improving community relations. Challenging lateral violence in a firm but respectful way, without retaliating, was empowering. Lastly, knowledge holders “turned a negative into a positive” (Clark et al., 2017, p. 117) by reframing a negative experience into a positive one. This reframing strategy took “the power out of the words” for the victim, making it easier for them to cope with their experiences (Clark et al., 2017, p. 118).

Few other strategies for coping with lateral violence have been identified. Bailey (2020) and Waterworth et al. (2014) reported that Indigenous people disengage from other Indigenous people or Indigenous spaces in order to protect themselves from lateral violence. In addition, Bennett (2014) found that some Indigenous people chose to hide their Indigenous heritage or not identify as Indigenous to avoid lateral violence. However, these studies did not explicitly investigate the coping strategies employed for lateral violence. Limited literature exists on the strategies Aboriginal people use for coping and dealing with lateral violence.

### Current study

Given the prevalence and impacts of lateral violence on Aboriginal peoples’ lives, further investigation into the coping strategies employed to navigate lateral violence is warranted. This research aimed to gain a better understanding of how Aboriginal Australians cope and deal with lateral violence. The research aim was intentionally left broad in order to capture a wide range of responses not limited to the research team’s knowledge in this area.

## Methods

### Research framework and team

This study was conducted using an Indigenous Research Paradigm (Wilson, 2008). Central to this paradigm is relational accountability, “being accountable to your relations” (Wilson, 2008, p. 77). It is, therefore, essential in Aboriginal-led research that the team introduce themselves in order to provide accountability and acknowledge the position from which they research (Kovach, 2009). The lead author, Theoni Whyman, is a Paakantji scholar with a background in qualitative health research, psychology, and public health. Cammi Murrup-Stewart is also an Aboriginal scholar with a social sciences background. Laura Jobson and Adrian Carter are non-Aboriginal scholars with experience in cross-cultural health and psychology. Uncle Michael Young is a Paakantji/Parrintji community Elder. He, and two other Elders, played a role in various stages of the research including conceptualisation, research design, and interpretation of findings.

Ethics approval was gained from Monash University Human Research Ethics Committee (ID:18189). Our research adhered to the six core values in conducting ethical research with Aboriginal peoples (Laycock et al., 2011; Whyman et al., 2021). Additional details about the methodological underpinning of the research and other background details are outlined elsewhere (see Whyman et al., 2021).

### Knowledge holders

Seventeen Aboriginal knowledge holders (term used instead of “participants” see AHRC, 2020) chose to share their knowledge for this study. Knowledge holders were aged 18 to 65 years, with most being in the 25 to 29 age group. Knowledge holder demographics are summarised in Table 1. Knowledge holders identified as male (*n* = 9) and female (*n* = 8). Almost two-thirds of knowledge holders were staff or students at an Australian university. The remaining third were employed in various professions. Knowledge holders resided in south-east Australia.

**Table 1.** Knowledge holder demographic information.

Pseudonym	Gender	Age range
Jannali	F	25–29
Waru	M	45–49
Ben	M	45–49
Jessika	F	25–29
Kalina	F	30–34
Alinta	F	25–29
River	F	18–24
Shareena	F	40–44
Marlon	M	18–24
Jaxon	M	18–24
Jarrah	M	25–29
Ben	M	45–49
Narelle	F	50–54
Uncle Pete	M	55–59
Josh	M	35–39

Recruitment began in February 2020 using emails and social media adverts. Those who expressed interest were invited to attend an individual yarn or yarning circle (described below) to discuss lateral violence. All knowledge holders were informed of the purpose of the research and provided informed consent. A verbal definition of lateral violence was provided at the beginning of all yarns. This was followed by a reflection on various aspects of lateral violence by knowledge holders. Knowledge holders received a $40 reimbursement for their time.

### Yarning methodology

Yarning is often used by Aboriginal researchers to investigate a topic in Aboriginal communities (Bishop et al., 2012; Kilcullen et al., 2017; Murrup-Stewart et al., 2020). When used correctly, yarning is a culturally appropriate method that can facilitate stories or information being shared between two or more people (Bessarab & Ng’andu, 2010; Lin et al., 2016; Murrup-Stewart et al., 2022). Yarning is a rigorous research method for collecting knowledge based on an Aboriginal epistemology. A yarning circle occurs when two or more people engage in yarning and consists of “deep listening, self-reflection and accountability” (Ballangarry, 2015, p. 5). For more detail regarding how yarning was used in this study, see Whyman et al. (2021).

Yarns occurred as two in-person yarning circles (*n = 7*), and ten individual yarns. Due to COVID restrictions, individual yarns were face-to-face (*n* = 2) or online (*n* = 8). Yarns were audio-recorded and transcribed professionally. Knowledge holders reviewed and agreed to the use of their quotes for all publications.

### Yarn analysis

A combination of both thematic analysis (Braun & Clarke, 2006) and yarn summaries were used to analyse the yarns. TW created summaries of each yarn, including how TW and each knowledge holder knew each other and their yarn about lateral violence. The summaries allowed for each person’s knowledge to be kept in context. This was important in maintaining an Aboriginal epistemology as thematic analysis (Braun & Clarke, 2006) breaks down data into codes and themes, potentially separating knowledge from the context in which it was shared. Yarn summaries were approved by knowledge holders and formed a preliminary analysis that incorporated both the facilitator and the knowledge holder’s perspective of meaning.

Thematic analysis of the yarn transcripts followed Braun and Clarke’s (2006) six steps: (1) data familiarity, (2) generate initial codes, (3) search for themes, (4) review of themes, (5) define and name themes, and (6) produce a report. The generation of codes and themes occurred iteratively and inductively, allowing for new themes to emerge. The whole research team was included in discussions of codes and themes and contributed to producing this manuscript. Yarn summary and thematic analysis findings were combined and reported together to provide a fuller description of the results.

## Results

As knowledge holders reflected on their strategies for preventing or coping with lateral violence, many talked about: (1) changes in attitude towards lateral violence, (2) connecting with others, (3) connection to culture and community and improving wellbeing, (4) education, and (5) systemic strategies. The first theme, changes in attitude towards lateral violence, encompasses two subthemes, (i) identity and self-acceptance, and (ii) power in dealing with lateral violence.

### Changes in attitude towards lateral violence

Knowledge holders mentioned that their strategies for coping had changed as their attitude towards lateral violence had changed over time. Jannali, who experienced lateral violence within her family, revealed that,

The way that I’ve dealt with lateral violence has changed over time. It used to really, really get to me, like I remember as a kid being really affected by things that my mum would say, like that really got to me and I would cry a lot about that because I wanted to belong to a community who accepted me and loved me … As I’ve gotten older I’ve learnt why that is and now I can process it and understand where my family is coming from, so it’s a much more intellectual way of dealing with it rather than an immediate, emotional response.

In reference to his youth, Waru said,

The ways that I coped with lateral violence was overcompensating and listening too much to the judgements of others, and, how could I say, tried too hard to fit into being an “authentic” black person according to these people who were discussing identity … My strategies around dealing with lateral violence have changed as I’ve grown and developed as an Aboriginal man and in my own ways of looking at identity.

Ben also reflected on his changes in dealing with lateral violence,

Back in my younger days it’s like you crack [fight] them and then … you ask questions later. But when you get older you just, you learn to … I’ve learned to sort of just block it out and just walk away.

#### Identity and self-acceptance

Some knowledge holders coped with negative comments by having developed a strong sense of themselves, their identity, and by accepting who they are. Waru reflected,

I’ve been in community long enough now to have formed a stronger identity. My identity is stronger and more solid and I have a much stronger understanding of my cultural identity … Knowing that and accepting that has been a great – it’s really liberated me from worrying so much about people’s comments. I really cope by just accepting that this is who I am and this is my community and people who know me.

In a similar vein, Jessika explains how her acceptance gave her confidence,

I had to build up coping mechanisms and it comes mainly through self-assurance of I know. I know my heritage so if somebody else tells me that I don’t or that I’m not enough or whatever, I’m like nobody else can say anything worse to me than what I say to myself.

Kalina noted that it can be “hard because not everyone knows [who they are]- everyone’s on their own. Some people have a strong sense of identity, some people may be a bit less strong in that”. For those who are less sure of themselves and their Aboriginality, Jessika believes that some “sort of confirmation or external validation for people would be really good” in helping them cope with lateral violence. She also reinforces that “if you identify as Indigenous then you’re Indigenous”.

A common strategy for coping with lateral violence was the recognition that lateral violence had little to do with whoever was being targeted. Some knowledge holders tried to look for an explanation for laterally violent behaviours. In Alinta’s experience of lateral violence at work, she realised that “it’s actually that I’m very good at my job and the threat is that I could surpass [the perpetrator] in that space. That if I put myself in his shoes, that would be quite threatening”.

In some instances, River recognises that “[lateral violence is] usually coming from like a dark place of trauma versus – yeah, like so I kind of give them the benefit of the doubt”. Jannali adds that “I can process it and understand where my family is coming from, so it’s a much more intellectual way of dealing with it rather than an immediate, emotional response”. Similarly, Shareena says “I’ve probably intellectualised it a little bit so as to not take it so personally”. Waru also does not take laterally violent comments personally. Instead, he has the view that,

What people think of me is none of my business. I guess as I’ve gotten older I just really care less, basically, about what people think of me. That’s one way that I now deal with lateral violence, that I just make a conscious decision to not want to really – I’m not interested in what other people say about me.

#### Power in dealing with lateral violence

Some knowledge holders mentioned power when yarning about coping with lateral violence. Shareena recognised that

The older I got, I would see questions about: who are you, who’s your mob, where are you from, as a challenge. Whereas someone described it to me as, with the power of it, to say that person is trying to make a connection with you. So, I flipped the power into my favour to say, oh yeah, interesting my connection to you. So, it took the heat out of it for myself.

Similarly, Jessika acknowledged how powerful lateral violence can be and how her coping strategy assisted in decreasing the power of lateral violence, “you give something power by keeping it inside. If you can have a laugh with someone about the ridiculousness of what somebody said, then it would lose power”.

Humour was identified as a strategy for coping with experiences of lateral violence. Jannali says, “A lot of the time when [lateral violence] happens I’ll just joke it off”. Likewise, Jessika likes to “have a laugh with someone about the ridiculousness of what somebody said”.

### Connecting with others

Relationality is about the connection people have with others. When knowledge holders sought support from family, friends, community, or services maintaining or building relationships was used to cope with lateral violence. In this way, people chose to “spend time with people who I value” (Kalina).

Many knowledge holders debriefed with someone in order to deal with lateral violence. River noted “having a yarn with some of my friends who kind of can relate to it … talking to people who’ve kind of had similar experiences is also helpful”. Marlon adds, “yarning and consulting with people that I know and respect and trust to give me good advice … I’ve always found that that’s the best way that I’ve coped with [lateral violence]”.

While some knowledge holders relied on family and friends for support, others sought the support of Elders, other members of the Aboriginal community and community organisations. “I have a couple of key members of community that I’ll have a chat with, basically. Have a yarn and be like, so this happened today” (Jessika). Jessika believed community members can provide valuable mentorship for dealing with lateral violence,

I think [support] comes from basically buddying up with a senior member of Community … so that you have someone who has been through this and can sort of teach you or coach you or even just support you when you’re going through these moments. (Jessika)

River noted that,

Referring [others] to groups and things. Like, Koori Youth Council [an Aboriginal youth advocacy group] is a place that I kind of try to send people to because … you get to know other people in the area and you get to know people [across Victoria] which means you kind of start to build that community around yourself.

Other knowledge holders considered professional forms of support. Jannali notes that “I can actually talk to my psychologist or my psychiatrist about [lateral violence], that sort of stuff”. Jaxon concurs that “Indigenous psychologists can probably have a large role to play in lateral violence”.

There may be some inherent challenges when connecting with others who have acted in a laterally violent manner. Some knowledge holders wanted to talk to the person who perpetrated the lateral violence, although this was often done with caution. Marlon takes time “to process and [cognise the lateral violence] and then come back to the person and talk it through with them”. Similarly, River tried to advocate for the victim while talking to the person who perpetrated the lateral violence explaining, “oh that’s not a good thing to say; think about the impact that’s going to have on them”.

While speaking to others was commonly used by knowledge holders to cope with lateral violence, others recognised the challenges that this could bring to Aboriginal people. In a work setting, Alinta mentioned that while she escalates issues of lateral violence to a line manager, this can reflect poorly on Aboriginal people,

So I’ve just started putting it up to people higher than me, which I didn’t like doing because they’re not Indigenous people and I don’t want those people in those positions to think that we can’t handle ourselves as mob when we’re so few within this organisation. But [lateral violence is] not something that we can work out on our own.

Similar sentiments are expressed by Jarrah,

The problem with it that I have that I see with lateral violence happening in the public is that the media and other white people and White Australia take it on and they use it to show, or they use it to display, why Aboriginal people aren’t responsible and they shouldn’t get power … shouldn’t have self-determination, they shouldn’t have a voice to the government, et cetera. [Lateral violence is] a big issue within Aboriginal Australia because it, essentially, can be used as a weapon by the colonial system against us.

Finally, relationality can also be about choosing not to have a connection with some people: “I choose who I hang out with … I don’t hang out with people – and I limit my association with – people who are so judgemental around cultural politics” (Waru). Similarly, Ben stated he “stay[s] away from people that are disrespectful and that try to use lateral violence towards me”.

### Connection to culture and community and improving wellbeing

Knowledge holders talked about how lateral violence is “symptomatic of loss of identity, loss of pride, lack of connection to culture and Country” (Narelle). Narelle elaborated, saying, “it’s symptomatic … of hurt and of trauma and I guess [addressing lateral violence is] about rebuilding that strength and rebuilding spirit”. Many knowledge holders agreed that strengthening one’s connection to culture, community, and identity would prevent instances of lateral violence. While discussing ideas for preventing lateral violence, Kalina asked rhetorically, “If people are able to strengthen other parts of themselves hopefully that can help and be aware … like how do we support people’s wellbeing, how do people strengthen their cultural wellbeing, how do people strengthen their spirit?”

Many knowledge holders thought it was the role of community to support those being targeted by lateral violence. This could be done in multiple ways. Narelle explained it using an example from the education sector,

Aboriginal people being on the panel are interviewing [Aboriginal] youngsters, but the inference is that the students Aboriginality is being called into question … I see that as lateral violence … [Students are] coming and identifying with [Indigenous student engagement unit]. Therefore, I think that we then are their community. I think [Indigenous staff] have an obligation to actually help develop that pride and strength and their culture and provide those links where we can … That’s the very stuff that’s going to actually help them resist the bullying and the colonisation that exists within the system.

Here, Narelle saw the role of developing a sense of community and culture in buffering the effects of lateral violence. Shareena experienced this benefit first-hand. She explained, having experienced lateral violence in the workplace and how her connection to community had protected her,

[A colleague had] felt challenged in the workplace, and then tried to undermine [me], you know, to [justify] what they were doing to try to undermine me in community, but because I live and work in this community and am established here generations deep and that person was not, it didn’t work. But that was the only protection that I had.

Jessika believed that community should also be supportive of Aboriginal people who do identify,

As long as someone is engaging [in culture] then we can build up that attitude of … being supportive of each other but also just making pathways that are easier for people to identify … not shaming people if they don’t know who their mob are because there’s a reason. [for not knowing]

River agreed, “it’s up to community to try and support [victims] through that process, not kind of push them further away by saying these things which could be just off-handed comments but really affect the person who they’re directed towards”. To begin to address lateral violence, Waru said,

It’s going to take more and more Aboriginal people to really unpack identity and cultural identity from a social and historical lens and really start to tease out what identity and Aboriginal identity is and how lateral violence relates and how that impacts… I believe that it has to come from Aboriginal people where there are going to be more and more sophisticated discussions around the complexities of identity. I do think that will eventually help counter elements of lateral violence, but it will take time.

### Education

Many knowledge holders talked about raising awareness for lateral violence within communities: “it is about the awareness that we need to get out there and actually explain to them that [lateral violence] is unacceptable behaviour” (Uncle Pete). Similarly, Kalina added, “having an understanding of what they are doing is lateral violence and that it’s not okay and what the impacts are [could be useful]”. She elaborated, drawing attention to a need for healing: “if it is coming from a place of their own hurt and anger maybe they need to go on their own healing path and figure that out rather than putting it on others”. Such self-reflection may be brought to awareness during education around lateral violence.

The importance of education around lateral violence was made clear by Ben,

If we can teach Communities or people in the Community what lateral actually means, lateral violence, and what it represents and what it does to people, then people will have a little bit more understanding of what lateral violence is all about. Then they’ll realise that some little things that they’re doing and that are offending other people and hurting other people and degrading other people, and they’ll realise that they’re using lateral violence and they need to stop.

Jannali described the intergenerational impacts that education can have, “where it starts with us, it will be passed onto our kids, passed onto their kids, so in three generations our kids just know this stuff”. Suggested content for lateral violence education included behaviour change courses, learning coping strategies, learning how to resolve conflict healthily and increasing resilience.

However, Kalina noted that she had previously attended a lateral violence workshop and left feeling deflated,

I went to this event … and it was about lateral violence and I … just left feeling quite deflated. Because it’s kind of like, okay this happens but … It’s just another thing that we are having to deal with, another burden.

### Systemic strategies

Knowledge holders believed addressing lateral violence would require a systemic approach. As Kalina stated,

I feel like there’s only so much that can be – there’s certain things that you can do within a community but there needs to be probably broader changes that are beyond the Aboriginal community to address colonisation and the impacts and oppression. Ideally you would just resolve oppression and people will then not feel so powerless.

A suggestion for reducing lateral violence in the workplace came from Alinta, “the system should allow for two strong leaders who of both Aboriginal descent to be in a workplace like this. Rather than have, it pivoted one against the other, and there’s only one top position” (Alinta).

Other factors identified included social determinants of health such as improved employability, financial security (River), and including lateral violence in workplace policy documents “put it in your RAP [Reconciliation Action Plan], make it a requirement to address these issues like we address other racialised issues” (Alinta).

Other systemic strategies proposed included self-determination and increased access to resources. Marlon expressed deep thinking in this area,

I’d say promoting self-determination and giving our communities access to increased resources in a way that allows them to feel a sense of prosperity and cultural strength. That would promote resilience as well as not only reducing some of the things that I’ve seen, which is the inadequate control of resources by community members against other community members … because that would target supporting outcomes in terms of buffering the effects of lateral violence and reducing at least some of the potential risk factors for lateral violence, which would be, I imagine, poverty and oppression. Yeah.

Josh highlights just what a systems-wide solution could mean for Aboriginal Australians,

The severe disadvantage we are at as a people is automatically perpetuating the breeding ground of … lateral violence. I guess that’s why I feel so many people look towards something like a treaty process or some people speak of constitutional recognition. Myself, sovereignty. I dream of sovereignty because it will put our people back in a position of equity. A position to potentially have access to resource or access to liberal connections to one’s own country and hopefully be able to build a life that is purely self-determined.

## Discussion

Lateral violence is a harmful interpersonal behaviour endemic among Aboriginal people. This study investigated the strategies Aboriginal people employ to cope with experiences of lateral violence. Knowledge holders revealed a deep understanding of their methods for coping and dealing with lateral violence. Strategies discussed were related to knowledge holders’ changing attitudes towards lateral violence, the way they connect with others, how their connection to culture and community can support wellbeing, education, and systemic strategies.

Knowledge holders’ connections to others, culture, and community were important in coping with lateral violence. This may be unsurprising given that strong connections to culture and community comprise good Social and Emotional Wellbeing (SEWB; Aboriginal conceptualisation of health and wellbeing) and when SEWB is supported, an individual may feel more resilient (Gee et al., 2014). In support of this, knowledge holders believed that connecting with the broader Aboriginal culture and community can buffer against the impacts of lateral violence and support one’s wellbeing. It was noted that connection to culture and community offered much needed support in a time where connections can be tested – as it can be one’s connections to other Aboriginal people, community, and culture which cause an individual to be targeted for lateral violence in the first place (Whyman et al., 2021). It seems strengthening these connections would serve two purposes, not only to cope with lateral violence but to also improve wellbeing. Other research has similarly found that when Aboriginal people connect to their culture and community their wellbeing improves (Murrup-Stewart et al., 2021). Therefore, resources should be dedicated to increase SEWB among Aboriginal people to improve coping with lateral violence.

Discussions about identity were related to developing a sense of oneself and accepting oneself as a strategy to cope with lateral violence. It became apparent that a strong sense of identity was important for overcoming stereotyped notions of an Aboriginal person and overcoming challenges to one’s identity. But how to develop a strong sense of one’s Aboriginality in the first place? It was noted that this may be difficult when an individual is less confident in their identity and culture. Thus, this strategy may only be available to those individuals who already have a strong identity. However, developing a strong sense of identity should be prioritised as it has been identified that a strong identity and strong spirit vaccinates against lateral violence (Gorringe et al., 2011).

Knowledge holders reflected that little is still known about lateral violence at a community level. They echoed previous research in stating that education strategies could improve community knowledge and reduce lateral violence (Clark et al., 2017). However, it was emphasised that workshops alone are not adequate. Rather, lateral violence education should be provided in the broader context of ongoing trauma and systemic causes of lateral violence (Whyman et al., 2021) and in consideration of the emotional load that the Aboriginal community bears (Dudgeon et al., 2014). This, in addition to insights shared by knowledge holders here and in Clark et al. (2017), would be beneficial for Aboriginal communities experiencing lateral violence.

Focusing on systemic strategies to reduce lateral violence may have the most impact. Many knowledge holders discussed broader systemic strategies that could be adopted aligning with other research concerning Aboriginal wellbeing (Dudgeon et al., 2014; Verbunt et al., 2021; Watego et al., 2021). Systemic strategies posed to deal with lateral violence included self-determination and adequate resourcing of communities and workplaces. It was thought that self-determination and adequate resourcing may act as a mechanism for communities to assert their needs, promote cultural strength and resilience, and take back some of their power. This may also strengthen cultural identity. Self-determination has been found to have a positive impact on Aboriginal health and wellbeing (Verbunt et al., 2021) as well as being a guiding principle underpinning SEWB (Gee et al., 2014). Facilitating self-determination in Aboriginal communities will therefore improve SEWB, reduce instances of lateral violence and heal communities from the powerlessness and oppression which created lateral violence in the first place (Whyman et al., 2021).

### Limitations and future directions

Several considerations are worth noting. First, these findings represent a limited group of Aboriginal people and care should be taken when applying these findings to other groups. Second, the dearth of literature in the field of lateral violence means very little is known in the academic space. Although this study expanded our understanding, without further research the connection between identity and lateral violence remains somewhat unclear. The role of identity in lateral violence could benefit from more evidence. Finally, much of this research took place online during COVID-19 restrictions. Holding some yarns virtually may have had an impact on the knowledge holders’ engagement. However, a comparison of the quality and length of face-to-face yarns and online yarns was found to be equivalent.

## Conclusion

This paper highlighted strategies for coping and dealing with lateral violence, which is crucial to examine as lateral violence has been identified as endemic among Aboriginal communities (Frankland, 2008). The proposed strategies are strengths-based and community-focused. They highlight the strength of Aboriginal people and their cultures, in that fostering connections to these domains of SEWB can drastically improve the wellbeing of individuals and the overall community. This, in conjunction with truly self-determined Aboriginal communities, has the capacity to heal all Aboriginal people.

## Data Availability

Participant data from this study is not available to be shared more widely than they are through reports such as this manuscript.
